# An Economic Analysis of Cell-Free DNA Non-Invasive Prenatal Testing in the US General Pregnancy Population

**DOI:** 10.1371/journal.pone.0132313

**Published:** 2015-07-09

**Authors:** Peter Benn, Kirsten J. Curnow, Steven Chapman, Steven N. Michalopoulos, John Hornberger, Matthew Rabinowitz

**Affiliations:** 1 Division of Human Genetics, Department of Genetics and Genome Sciences, University of Connecticut Health Center, Farmington, CT, United States of America; 2 Illumina Inc., San Francisco, CA, United States of America; 3 Natera Inc., San Carlos, CA, United States of America; 4 Cedar Associates, Menlo Park, CA, United States of America; 5 Cedar Associates, Menlo Park, CA, United States of America; 6 Stanford University, Stanford, CA, United States of America; Institut Jacques Monod, FRANCE

## Abstract

**Objective:**

Analyze the economic value of replacing conventional fetal aneuploidy screening approaches with non-invasive prenatal testing (NIPT) in the general pregnancy population.

**Methods:**

Using decision-analysis modeling, we compared conventional screening to NIPT with cell-free DNA (cfDNA) analysis in the annual US pregnancy population. Sensitivity and specificity for fetal aneuploidies, trisomy 21, trisomy 18, trisomy 13, and monosomy X, were estimated using published data and modeling of both first- and second trimester screening. Costs were assigned for each prenatal test component and for an affected birth. The overall cost to the healthcare system considered screening costs, the number of aneuploid cases detected, invasive procedures performed, procedure-related euploid losses, and affected pregnancies averted. Sensitivity analyses evaluated the effect of variation in parameters. Costs were reported in 2014 US Dollars.

**Results:**

Replacing conventional screening with NIPT would reduce healthcare costs if it can be provided for $744 or less in the general pregnancy population. The most influential variables were timing of screening entry, screening costs, and pregnancy termination rates. Of the 13,176 affected pregnancies undergoing screening, NIPT detected 96.5% (12,717/13,176) of cases, compared with 85.9% (11,314/13,176) by conventional approaches. NIPT reduced invasive procedures by 60.0%, with NIPT and conventional methods resulting in 24,596 and 61,430 invasive procedures, respectively. The number of procedure-related euploid fetal losses was reduced by 73.5% (194/264) in the general screening population.

**Conclusion:**

Based on our analysis, universal application of NIPT would increase fetal aneuploidy detection rates and can be economically justified. Offering this testing to all pregnant women is associated with substantial prenatal healthcare benefits.

## Introduction

There are over 4 million pregnancies annually in the United States, the majority of which undergo fetal aneuploidy testing [1]. There are many options, with test selection largely dependent on a woman’s prior risk, pregnancy stage, access to healthcare services, cost, and/or patient preference. Although the American Congress of Obstetricians and Gynecologists recommends offering diagnostic testing to all women [2], many initially choose prenatal screening. Historically, first trimester screening has involved maternal serum marker evaluation plus ultrasound [2], while those entering prenatal care in the second trimester usually received maternal serum markers and some received sonographic marker evaluation. The newly-available non-invasive prenatal tests (NIPTs) offer higher detection rates and lower false positive rates than pre-existing screening methods [3]. Following validation studies in high-risk patients, NIPT was endorsed for use in high-risk women by a number of professional bodies [4, 5]. Recent data has shown that NIPT is also effective in women with low prior risk [6–10]. The American College of Medical Genetics and Genomics guideline does not specify prior risk as a factor for offering NIPT [11]. A recent position statement from the International Society for Prenatal Diagnosis recognizes that NIPT can be offered as a first line prenatal screening test for all women [12]. However, a major hurdle preventing test expansion into the general pregnancy population stems from economic concerns.

Previous studies have evaluated the circumstances where NIPT is economically justifiable [13–26]. However, these studies did not fully consider the diversity of test options utilized in the US and only one study has considered disorders other than trisomy 21 [26]. In this study, we determine the cost effectiveness of replacing conventional screening approaches with NIPT in a general US screening population taking into consideration these additional factors.

## Methods

We constructed a decision-analysis model to evaluate the economic value of fetal aneuploidy screening in the annual US general screening population Screening and invasive test utilization, miscarriage, and pregnancy termination rates were all considered.

Development of the model is described in more detail in S1 Methods. Briefly, modeling was based on a theoretical cohort of 3,952,841 live births, which represented the number of births in the United States in 2012 [1, 27]. For this population, the number of births at each maternal age at delivery was known; 14.9% were to women aged 35 or older. The model was based on 70% of pregnant women in the US receiving some type of fetal aneuploidy prenatal screening; this figure was based on survey data collected prior to the introduction of NIPT [28]. We conservatively assumed that the screening utilization rate would remain at 70% with NIPT. Thus, for the purposes of the economic analysis, patients that do not undergo any screening are considered cost neutral and can be excluded from the model.

The screening strategies compared were conventional screening versus NIPT with cell-free DNA (cfDNA) analysis. This economic analysis was designed to: (a) assess the value of NIPT as a replacement for conventional screening approaches; (b) determine the number of additional fetal trisomy 21, 18, and 13 and monosomy-X cases prenatally detected; (c) estimate the reduction in the number of affected births, and; (d) assess the reduction in the number of invasive procedure-related unaffected fetal losses. The economic analysis considered the cost of all components of screening, diagnosis, counseling, and pregnancy intervention or medical costs associated with an affected birth. The result was expressed as the per-case charge for NIPT that would be cost neutral to the healthcare system. Monosomy-X was included because there is data to show that NIPT can be effective in screening for this disorder (S2 Table) and because it is common practice in the US to provide the testing for sex chromosome abnormalities. However, we excluded other sex chromosome abnormalities from the analysis due to lack of data on screening performance and the extent of follow-up of cases with high-risk results. The additional value associated with some tests (e.g. maternal age alone, alpha fetoprotein [AFP], and nuchal translucency [NT]) for the detection of abnormalities other than the previously defined set of aneuploidies was not considered. Some aneuploidies associated with major malformations might be initially suspected through an early dating ultrasound and these were not considered. Similarly, the ability for NIPT to detect additional conditions such as microdeletion syndromes was not evaluated. Other costs of screening that can be encountered in clinical practice, non-medical costs that might be incurred by patients, the value of early reassurance to those women who receive low risk results, and the cost of any ancillary testing were excluded in this analysis. For example, the additional expense associated with chromosomal microarray analysis to detect small imbalances (in addition to aneuploidy) was excluded.

The first trimester “Combined test” (measurement of NT together with maternal serum markers, pregnancy-associated plasma protein A [PAPPA] and free beta human chorionic gonadotrophin [hCG], at 12 weeks gestational age), sometimes in combination with additional second trimester screening tests, is a recommended approach for providing Down syndrome screening to women of all ages [2]. We therefore based our analyses primarily on women entering screening in the first trimester. The model took into consideration the Combined test’s efficacy for the detection of non-mosaic fetal Down syndrome, trisomy 18, trisomy 13, and monosomy-X. Additionally, this analysis considered the proportion of women: 1) subsequently receiving second trimester serum screening (i.e. integrated or sequential screening); 2) electing follow-up invasive testing (amniocentesis or chorionic villus sampling [CVS]); 3) electing pregnancy termination, and; 4) experiencing spontaneous pregnancy loss. Recognizing that a large number of women do still receive conventional screening based on the second trimester “quadruple test” (maternal serum AFP, hCG, unconjugated estriol, and inhibin-A) alone, we also constructed a subsidiary economic model that included varying proportions (up to 100%) of women entering screening in the second trimester.

The performance of conventional screening approaches was determined through multivariate simulations using SURUSS statistical parameters [29] (see S1 Methods and Table 1). The performance of NIPT for each aneuploidy was based on compiled data from validation studies (Table 1, S2 Table). Test adoption practices used in the model were derived from published studies or, if rates were unavailable, using consensus data obtained through survey of obstetricians currently providing prenatal screening and diagnosis. Full details of these procedure rates are presented in S1 Methods.

**Table 1:** Aneuploidy incidence rates and performance of conventional screening approaches in the first and second trimester for a general population.

	First Trimester	Second Trimester
**Trisomy 21**		
Prevalence	1/365	1/398
*Conventional Screening* ^^^		
Sensitivity	85.3%	84.1%
Specificity	95.2%	92.5%
*NIPT* *		
Sensitivity	99.3%	99.3%
Specificity	99.9%	99.9%
**Trisomy 18**		
Prevalence	1/1208	1/1487
*Conventional Screening* ^^^		
Sensitivity	95.0%	73.5%
Specificity	99.7%	99.8%
*NIPT* *		
Sensitivity	96.8%	96.8%
Specificity	99.9%	99.9%
**Trisomy 13**		
Prevalence	1/3745	1/4195
*Conventional Screening* ^^^		
Sensitivity	94.5% ^†^	16.4% ^‡^
Specificity ^§^	-	-
*NIPT* *		
Sensitivity	87.2%	87.2%
Specificity	99.8%	99.8%
**Monosomy X**		
Prevalence	1/1291	1/2340
*Conventional Screening* ^		
Sensitivity	75.0%	54.1%
Specificity ^§^	-	-
*NIPT* *		
Sensitivity	89.5%	89.5%
Specificity	99.8%	99.8%

^^^ Conventional screening based on maternal age, nuchal translucency, maternal serum pregnancy-associated plasma protein A [PAPPA] and free beta human chorionic gonadotropin [hCG] at 12 weeks gestational age.

* NIPT sensitivity and specificity was based on pooled data from 19 published studies (see S2 Table); NIPT sensitivity and specificity was considered to be independent of pregnancy stage and maternal prior risk.

^†^ First trimester sensitivity for trisomy 13 screening is based on the algorithm developed for trisomy 18 screening [30].

^‡^ Second trimester sensitivity for trisomy 13 screening is assumed to be equal to the proportion of trisomy 13 affected pregnancies serendipitously identified as a false positive in Down syndrome and trisomy 18 screening.

^§^ No specific screening protocols exist for trisomy 13 and monosomy X.

The cost estimates used in the baseline model analysis included those associated with office visits, genetic counseling, screening, invasive testing, termination procedures, and lifetime costs of delivering an affected child (S1 Table). Where possible cost estimates were derived from the literature in addition to the 2014 Centers for Medicare and Medicaid Services (CMS) Fee Schedules [31, 32]; all costs estimates were adjusted for inflation to 2014 US Dollars using the Medical Care Component of the Bureau of Labor Services Consumer Price Index. Centers for Medicare and Medicaid Services (CMS) rates were increased by 20 percent to estimate the expenditure from the perspective of private or commercial payers, as described previously [14]. The final cost of a particular test or procedure is the sum cost of all individual components. All costs were reported using 2014 US Dollars.

We also designed the analysis to show how costs would change when replacing conventional screening with NIPT in a study population restricted to women considered high-risk because of advanced maternal age (AMA; ≥35 years). The sensitivity analysis evaluated test costs from 40% below to 20% above the baseline and lifetime costs from 20% below to 20% above the baseline. To determine how differing practice patterns contribute to the value of NIPT, the sensitivity analysis evaluated key practice patterns for a range of clinically relevant inputs such as the timing of screening entry, invasive confirmation rates, and termination rates.

This study did not involve use of clinical records or any other patient information requiring approval by an ethics committee/institutional review board or data protection agency/commissioner.

## Results

Based on our model, the adoption of NIPT in clinical practice by all pregnant women who receive aneuploidy screening in the US results in a per-case NIPT value of approximately $744. This number reflects the average per-case cost offset between the two screening approaches; a charge higher than $744 would represent a net increase in overall expenditure and a lower charge would constitute a savings to the system. This $744 figure includes costs for subsequent additional screening and follow-up diagnostic testing ($86), genetic counseling ($3), lifetime costs associated with affected births ($286), and the current cost of conventional screening approaches ($369). The cost of conventional screening ($369) is a build-up of all prenatal screening costs, and accounts for the proportion of patients billed for/receiving each individual cost component (see S1 Methods), and includes: $70 for a separate office visit for NT, $10 for genetic counseling, a $76 charge for ultrasound, $17 for genetic sonograms, $147 for NT, and $49 for serum analytes. The corresponding cost offset for NIPT offered only to women of advanced maternal age (AMA, ≥35 years) is $1474.

For women entering screening in the second trimester, the per-case value of NIPT was estimated to be $486. This lower figure reflects the fact that second trimester screening tests are less expensive than first trimester tests, and these women do not receive additional screening tests as part of sequential or integrated protocols (S1 Table). The somewhat inferior detection rate and higher false-positive rate of second trimester screening is also incorporated into this latter estimate. Table 2 shows estimates for various mixtures of first and second trimester screening. In contrast to conventional screening, the timing of entry for NIPT had little impact on NIPT value.

**Table 2:** Model inputs and economic value of NIPT for fetal aneuploidy screening.

Model Input Variable	Baseline Value	Range	NIPT Range^^^	Reference
*Practice Patterns*				
Percent entering first trimester for conventional screening	100%	0%^¥^–100%	$486–744	[28]
Percent entering first trimester for NIPT	66%	0%^¥^–100%	$743–745	[6]; see text
Invasive testing uptake for conventional screen FPs	45%*	25%–100%	$738–747	[33]; see text
Termination Rates	65–90%^†^	0%–100%	$459–788	[34]
*Key Cost Variables* ^‡^				
Cost of first trimester screening	$369	$222–443	$597–818	[15, 16]; see text
Cost of sequential screening	$136	$82–164	$714–759	[15, 16]; see text
Cost of invasive testing (amniocentesis/CVS)	$835 / $892	$501–1,070	$740–747	[35]; see text
*Lifetime costs of an affected child* ^§^				
Trisomy 21	$677,000	$541,600–812,400	$687–802^˅^	[36–38]; S1 Table
Trisomy 18	$29,307	$23,446–35,168	$687–802^˅^	[36–38]; S1 Table
Trisomy 13	$33,577	$26,862–40,292	$687–802^˅^	[36–38]; S1 Table
Monosomy X	$271,010	$216,808–325,212	$687–802^˅^	[36–38]; S1 Table

FP(s), false positives; CVS, chorionic villus sampling; NIPT, non-invasive prenatal testing

^^^ NIPT values that correspond to the range applied to each input variable. These values can be compared to the $744 value assigned to the set of baseline model inputs.

^¥^ Alternative scenario showing results when all initial screening is performed in the second trimester.

* Baseline invasive testing rates for true positives were 73% for trisomy 21 and 90% for trisomy 13, trisomy 18, and monosomy X. These rates were not changed when the invasive rate for false-positives was adjusted.

^†^ Baseline termination rates for trisomy 21, trisomy 18, trisomy 13, and monosomy X were 87%, 81%, 90%, and 65%, respectively [34].

^‡^ The range evaluated for cost variables was baseline minus 40% to plus 20%. The cost of first trimester screening ($369) was a buildup of all the individual components (see Results).

^§^ The range of lifetime costs of an affected child was baseline ±20%; the upper and lower range values for each indication were modified together.

^˅^Most of the variability is attributable to lifetime costs for Down syndrome (see text).

Most of the financial benefit of offering screening resides in the detection of fetal trisomy 21 rather than the other common aneuploidies. This is because trisomy 21 is the most common fetal aneuploidy and has a high survival rate, leading to high medical costs. The cost offset when trisomy 18, trisomy 13, and monosomy X are excluded is essentially unchanged at $744; financial benefits associated with the detection of these disorders are offset by the costs of testing.

The effect of variation in key inputs on the value of NIPT in a general screening population is shown in Table 2. As expected, decreasing termination rates was associated with a reduction in NIPT value. Screening costs, particularly first trimester screening, are also important variables; reimbursement levels do differ depending on the insurance carrier, and there are inter-regional differences. The range of +40% and -20% shown in Table 2 was chosen to cover these potential inter-district cost variations [32]. Changing the cost of the first trimester Combined test had the greatest had the value of NIPT; a 40% reduction in the Combined test was associated with a decrease in NIPT value of $147, and a 20% increase in the Combined test cost was associated with an increase in NIPT value of $74. Modification in the lifetime costs of an affected child up or down by 20% had a relatively small effect on the overall value of NIPT, increasing or decreasing the value by around $58, respectively.

Replacing conventional screening with NIPT would increase fetal aneuploidy detection rates, decrease the number of affected births, and lower the number of invasive tests and thereby reduce procedure-related losses of euploid fetuses (Table 3). We estimate that, replacing conventional screening with NIPT would increase the number of affected pregnancies prenatally detected by 5.9% (364/6149) in a high-risk population and 12.4% (1403/11,314) in the general screening population. Replacing conventional screening with NIPT would reduce the number of affected births by 29.9% (604/2017) and 33.4% (1213/3,629) in the high-risk and general screening populations, respectively. NIPT is associated with a 71.7% (19,037/26,555) and 60.0% (36,834/61,430) reduction in the number of invasive tests performed in the high-risk and general screening populations, respectively. As a result, the number of procedure-related euploid fetal losses is also reduced with NIPT, with a 90.9% (100/110) reduction in the high-risk population and a 73.5% (194/264) reduction in the general screening population.

**Table 3:** Comparison of clinical outcomes from baseline analysis of the two screening approaches in a general screening population.

	Conventional Screening	NIPT
***Trisomy 21***		
Affected pregnancies screened *	7836	7836
T21 affected with positive result	6687	7783
T21 births averted ^†^	2901	4097
Invasive tests ^‡^	53,813	9010
Procedure-related euploid losses	246	13
***Trisomy 18***		
Affected pregnancies screened *	2364	2364
T18 affected with positive result	2246	2288
T18 births averted ^†^	426	436
Invasive tests ^‡^	5604	4282
Procedure-related euploid losses	18	12
***Trisomy 13***		
Affected pregnancies screened *	763	763
T13 affected with positive result	721	665
T13 births averted ^†^	293	268
Invasive tests ^‡^	614	4624
Procedure-related euploid losses	0	20
***Monosomy-X***		
Affected pregnancies screened *	2214	2214
MX affected with positive result	1660	1981
MX births averted ^†^	9	41
Invasive tests ^‡^	1399	6680
Procedure-related euploid losses	0	25
***All aneuploidies combined***		
Affected pregnancies screened *	13,176	13,176
Affected with positive result	11,314	12,717
Affected births averted ^†^	3629	4842
Invasive tests ^‡^	61,430	24,596
Procedure-related euploid losses	264	70

NIPT, non-invasive prenatal testing

* 70% of total affected pregnancies (30% receive no screening).

^†^ Assuming termination rates for trisomy 21, trisomy 18, trisomy 13, and monosomy X of 87%, 81%, 90%, and 65%, respectively [34]; excludes spontaneous fetal losses.

^‡^ Invasive tests (amniocentesis or chorionic villus sampling) performed in true positives and false positives.

## Discussion

Our study shows that NIPT, as a universal prenatal screening test, can be introduced without increasing the net cost to the US healthcare system. The baseline model was considered to be the most appropriate estimate since it considered NIPT as an alternative to first trimester screening, which is generally accepted as the optimal time for testing. In the baseline analysis, NIPT led to increased fetal aneuploidy detection, and fewer invasive tests and procedure-related euploid fetal losses. This was expected, given NIPT’s detection rates (87.2–99.3%) and false positive rates (0.1–0.2%). Overall, the cost-neutral value of NIPT was $744 in the general pregnancy population.

Multiple studies have evaluated the financial implications of offering NIPT versus conventional screening [13–26]. Most of these studies were restricted to screening for fetal Down syndrome and direct comparison is confounded by differences in the testing components for conventional screening and diagnosis, the costs assigned to these components, screening policies, utilization rates, and maternal ages within the population. However, some conclusions can be drawn. All studies found that there was an economic advantage in offering NIPT to women at high risk for fetal Down syndrome and several studies recognized the economic benefit of extending NIPT to additional women with intermediate Down syndrome risks established by conventional screening (contingent NIPT) [17, 19, 20, 25]. Some studies have assessed the marginal benefit afforded by NIPT under the assumption that conventional screening infrastructure will not be rapidly changed [24, 25]. This assumption may be appropriate for publically funded healthcare systems that are heavily invested in conventional approaches and where there is tight control over ad-hoc use of additional testing for risk refinement. However, this is not directly applicable to the US where patient choice and market-driven forces generally result in diversity in screening approaches and the subsequent management of follow-up testing. In this study and others that have considered the lifetime costs associated with individuals with Down syndrome, the benefit of providing NIPT as a primary screening test to all pregnant women has been recognized [15, 19, 24].

A recent study by Fairbrother et al. [26] suggested a unit cost of $453 (or less) would establish NIPT as the dominant primary screening strategy over conventional screening. This value was based on the performance of conventional screening for fetal Down syndrome, trisomy 18 and trisomy 13 but not monosomy-X. Many of rates and costs used by Fairbrother et al. differ from ours. Notably, the pre-test first trimester prevalence for trisomy 21 used in that study, 1/530, was not reflective of the current US population which we estimated to be 1/365 for 2012. Fairbrother et al. assumed all testing would be completed in the first trimester with no additional cost assigned to sequential screening or other second trimester procedures. In our analyses, we included these downstream costs. We also recognized the practical reality that some women who receive high-risk conventional screening results do not pursue any additional prenatal testing and this will add to the costs associated with affected births.

As with any decision-analytic model, there were some limitations that must be considered in the interpretation of the results. The modelled performance of conventional screening was based on SURUSS parameters [28] that appear to project better screening performance than meta-analysis derived parameters [38]. If the sensitivity and specificity in actual clinical practice were worse than modeled, the benefit of using NIPT would be larger. Similarly, actual performance of NIPT could differ from that achieved in clinical trials (S2 Table). We did not consider differences in test performance between different NIPT methodologies. Test failure rates and some indirect expenses were excluded (see Methods). NIPT test failures could result in additional costs because some of these may be associated with a low fetal fraction and an increased risk for chromosome abnormality, notably trisomy 13, 18, and triploidy [10, 39–41]. In these cases, the provision of additional screening tests, ultrasound examinations and invasive testing would add to costs. However, it is not currently possible to fully evaluate this extra cost because it will depend on the policy of the laboratory with respect to measuring fetal fraction, types and prevalence of these chromosome abnormalities, and the recommended testing follow-up for cases with a test failure due to low fetal fraction. We excluded microdeletion syndromes, which are now available with some NIPTs. A separate study of lifetime costs for these latter disorders, including the costs of beneficial postnatal treatment interventions, is needed. However, it is anticipated that inclusion of clinically severe microdeletion syndromes in an economic analysis would further increase the value of NIPT, and could provide significant cost savings to the healthcare system. In a clinical setting, there is considerable variation in gestational age at screening, use of sequential testing, genetic counseling, and ultrasound. Some of these variations are addressed in the sensitivity analyses (Table 2). It was assumed that the sensitivity and specificity of NIPT in lower risk women would be equivalent to that seen in high-risk women; there is increasing evidence to indicate that this is the case [5, 6]. We also did not analyze the consequences when some women, notably those at high a priori risk, by-pass specific components of conventional screening and proceed directly to NIPT or invasive testing. The analysis excluded the economic aspects of fetal abnormalities other than the defined aneuploidies that may be identified through testing. One of the principal concerns arising from a replacement of conventional screening with NIPT is that patients would no longer receive an NT assessment as part of routine prenatal care. As described in a recent study, the primary purpose of NT measurement is fetal aneuploidy risk assessment [42]. While NT may detect additional fetal abnormalities and congenital heart conditions, it should be noted that an enlarged NT is a relatively poor predictor for isolated congenital heart disease [42]. Most pregnant women will have a number of ultrasounds during which evaluations for fetal cardiac abnormalities might be made. If NIPT were to be introduced as a universally available prenatal screen, the value of NT would need to be assessed based on the detection of cardiac defects and other fetal abnormalities not associated with aneuploidy.

Importantly, the purpose of this analysis was not to place a monetary value on the life of an affected child. Rather, the objective was to make an economic assessment of NIPT, relative to conventional screening approaches, that could be used when establishing coverage and access policies. Ultimately, it is the parents’ acceptance of screening, diagnosis, and pregnancy management options that will determine the allocation of financial resources.

As data accumulates demonstrating the superior efficacy of NIPT over conventional screening for all pregnant women, it becomes increasingly clear that NIPT does need to be offered to all patients. Intangible benefits such as reduced patient anxiety through earlier and stronger reassurance further reinforce the need for universal NIPT availability. The analysis presented here indicates that this can potentially be achieved without adding to the overall cost of prenatal healthcare.

## Supporting Information

S1 Methods Supporting methods. (DOCX)

S1 Table Baseline cost variables. (DOCX)

S2 Table Assay performance with cfDNA NIPT. (DOCX)
