# Supplementary material for: An Economic Analysis of Cell-Free DNA Non-Invasive Prenatal Testing in the US General Pregnancy Population
Source: PLoS One. 2015 Jul 9;10(7):e0132313. doi: 10.1371/journal.pone.0132313 (PMC4497716; doi:10.1371/journal.pone.0132313)
Supplement: S1 Methods — (DOCX) [file pone.0132313.s001.docx]

**Supporting methods**

The maternal age-specific risk for Down syndrome was based on a logit curve [1] and the rates adjusted to the first or second trimester by allowing for naturally-occurring fetal loss [2]. Similarly, the risks for trisomy 18 and trisomy 13 were based on birth rates [3] adjusted for fetal losses [4]. There was also an adjustment in the model to allow for euploid spontaneous losses [5]. The risk for monosomy X was considered to be constant for all maternal ages, and estimates of prevalence were based on published rates [6-8]. After allowing for spontaneous fetal losses, the total number of pregnancies was 4,081,342 at 12 weeks gestation and, in the absence of any screening and intervention, 4,019,025 in the second trimester. Full-term estimates of prevalence for trisomy 21, trisomy 18, trisomy 13, and monosomy X in the whole population were 1/525, 1/4180, 1/7113, and 1/12,684, respectively. The prevalence of fetal aneuploidies in the first and second trimester are shown in Table 1.

The performance of conventional screening approaches for fetal Down syndrome was determined through multivariate simulations using established means, standard deviations, correlation coefficients, and extreme value truncation limits [9-11]. Monte Carlo methods were used to simulate 100,000 affected pregnancies and 100,000 unaffected pregnancies. Likelihood ratios were generated for each combination of marker results. These likelihood ratios were then combined with the maternal age-specific risk for a Down syndrome pregnancy. At each maternal age, the sensitivity and specificity were defined by the proportion of affected and unaffected cases, respectively, with a second-trimester risk exceeding 1:270. Finally, for the screening cohort as a whole, the overall sensitivity and specificity (Table 1) was determined by combining the age-specific sensitivities and specificities with the maternal age distribution of the modeled cohort [12]. For women undergoing sequential screening, a proportion of women will have their screening classification change from positive to negative, or vice versa, following the second trimester component of the testing; these proportions [13] were taken into consideration in the model.

Performance of the first trimester combined test for trisomy 18 screening was similarly assessed through multivariate simulations, using mean, standard deviation, and correlation coefficients; and truncation limits for NT [14] and PAPP-A [15]. The performance of second trimester serum screening tests for trisomy 18 was based on published statistical parameters [16]. Positive screening results were defined as those with a second-trimester risk greater than 1:100. For trisomy 13, first trimester screening statistical parameters were considered to be equivalent to screening for trisomy 18 [17] while second trimester cases would only be identified serendipitously because of false-positive results for Down syndrome and trisomy 18. Sequential screening was not used for trisomy 18 and 13; it was assumed that for those women who received both first and second trimester screening tests, the test follow-up would be based on the first trimester results alone. Conventional screening does not have specific screening algorithms for the identification of monosomy X. However, the model included the expectation that approximately 75% of affected pregnancies would be identified serendipitously by the first trimester combined test [18] and approximately 54% by second trimester screening [8].

For cell-free DNA (cfDNA)-based NIPT, assay performance was determined by pooling data from 19 published studies (Supporting Table 2), which includes studies from all four companies providing NIPT in the US (Natera, Inc., San Carlos, CA; Ariosa Diagnostics, San Jose, CA; Sequenom, San Diego, CA; Illumina, Redwood City, CA). Clinical studies with incomplete outcomes [19-26] or with known inclusion of previously-reported samples [27-29] were excluded.

Based on current NIPT referrals to Natera [26], we assumed 66% of patients would present for screening in the first trimester. For consistency between the two evaluated screening strategies, we assumed patients with a negative NIPT result would not receive an invasive test (amniocentesis or chorionic villus sampling [CVS]). Since a positive NIPT is associated with a very high probability of an affected pregnancy [26] and women undergoing testing would be counseled that confirmatory testing is essential for a definitive diagnosis [30] we based our model on a 90% invasive test rate. For prenatally ascertained and cytogenetically confirmed true positive cases, regardless of the screening strategy, we used reported pregnancy termination rates specific to each aneuploidy [31].

The model included the projection that 54% of patients receiving first trimester combined screening would also receive second trimester screening as part of an integrated or sequential protocol [32]. As with women receiving NIPT, we assumed that women with negative conventional screening results do not receive invasive testing. Because affected pregnancies will generally be associated with higher conventional screening risks and are more likely to present with ultrasound abnormalities, we incorporated different invasive testing utilization rates for affected (true positives) and unaffected (false positives) pregnancies. For true positives, we used a 73% invasive testing rate for Down syndrome [13], and a 90% invasive testing rate for trisomy 18, trisomy 13, and monosomy X because these affected fetuses are more likely to present with ultrasound abnormalities. An invasive testing rate of 45.1% was used for false-positives [33]. For those women who chose to proceed to invasive testing on the basis of positive first trimester combined test results alone, we assigned 76% to amniocentesis and 24% to CVS [34]. The sensitivity and specificity of invasive testing was taken to be 100%, and the modeled risk for a procedure-related fetal loss was 0.5% for both amniocentesis and CVS [35].

The cost of the first trimester combined test included the combined cost of an office visit, necessary analytes, and imaging. We also included the cost of genetic counseling in 18% of patients and a separate office visit for NT in 73% of patients; both figures are based on our practice survey results. For first trimester screening, a separate office visit for NT was included since NT requires a certified practitioner, which often results in a referral from their general practitioner or Ob/Gyn to a different location [36]. Second trimester screening and the second step of sequential testing did not incur fees for a separate office visit or pretest genetic counseling. It was assumed that a proportion of women considered high risk by maternal age or conventional screening results received a genetic sonogram. The proportion was based on an observation that 43% of women high risk on the basis of maternal age or first trimester screening also received a second trimester genetic sonogram [37] and 85% of maternal fetal medicine specialists used genetic sonograms to modify the risk for Down syndrome [38]. A similar proportion of high-risk women entering screening in the second trimester were also assumed to receive a genetic sonogram. The per-case cost of genetic screening was calculated as the cost of a genetic sonogram plus an office visit ($319) multiplied by the proportion of women electing screening (70%, [32]), the proportion of women having genetic sonograms to modify serum screening risk (43% * 85%), and the proportion of high-risk cases (17% and 19% for first and second trimester, respectively), to give a cost of $14 or $16 for genetic sonograms that are part of first trimester or second trimester screening, respectively.

The costs for post-test genetic counseling, confirmatory testing, and termination procedures were equivalent between the two screening modalities. It was assumed that prior to being offered an invasive procedure all patients with a positive screening result (NIPT, First Trimester Combined Test, Sequential, or Second Trimester Screening) were offered post-test genetic counseling [39]. The pricing for amniocentesis and CVS includes both laboratory and physician fees, with 20% of cases assumed to use rapid FISH in addition to standard cytogenetics. Laboratory fees did not include additional expenses that might be incurred through the use of chromosome microarrays instead of conventional karyotype analyses.

The model also considered the lifetime costs of caring for an individual with a severe genetic disorder. The lifetime costs associated with a trisomy 21 birth included both direct and indirect costs [40]. Trisomy 13 and trisomy 18 pregnancies that reach full term usually experience limited survival following birth. Thus, we conservatively estimated lifetime costs for trisomy 13 and trisomy 18 births as those limited to hospital expenses using a weighted mean of 2006 and 2009 data from the Healthcare Cost and Utilization Project - Kids’ Inpatient Database (HCUP-KID) [41]. In the absence of published studies reporting lifetime costs for a monosomy X birth, we incorporated the cost of human growth hormone (HGH) therapy between the ages of 2 and 14 years [42] which, according to the Turner Syndrome Foundation, ranges in cost from $10,000 to $40,000 per year. The cost of HGH therapy per monosomy X birth was conservatively estimated to be $12,000 per year for 12 years of treatment ($144,000). Additionally, 30% of patients born with monosomy X will be diagnosed and treated for congenital heart defects [42], so we also incorporated these diagnosis and treatment costs into the lifetime costs. These costs were calculated by taking the mean lifetime cost of relevant heart conditions [40] and multiplying this by the estimated prevalence of congenital heart defects (30% * $423,367) [42]. Combined, the lifetime cost of a monosomy-X birth was estimated to be $271,010 when adjusted for inflation. The lifetime costs assigned to each indication are shown in Table 1.

1. Morris JK, Mutton DE, Alberman E. (2002) Revised estimates of the maternal age specific live birth prevalence of Down's syndrome. J Med Screen 9: 2-6.

2. Savva GM, Morris JK, Mutton DE, Alberman E. (2006) Maternal age-specific fetal loss rates in Down syndrome pregnancies. Prenat Diagn 26: 499-504.

3. Savva GM, Walker K, Morris JK. (2010) The maternal age-specific live birth prevalence of trisomies 13 and 18 compared to trisomy 21 (Down syndrome). Prenat Diagn 30: 57-64.

4. Morris JK, Savva GM. (2008) The risk of fetal loss following a prenatal diagnosis of trisomy 13 or trisomy 18. Am J Med Genet A 146A: 827-832.

5. Hook EB, Topol BB, Cross PK. (1989) The natural history of cytogenetically abnormal fetuses detected at midtrimester amniocentesis which are not terminated electively: new data and estimates of the excess and relative risk of late fetal death associated with 47,+21 and some other abnormal karyotypes. Am J Hum Genet 45: 855-861.

6. Snijders RJ, Sebire NJ, Nicolaides KH. (1995) Maternal age and gestational age-specific risk for chromosomal defects. Fetal Diagn Ther 10: 356-367.

7. Hook EB, Warburton D. (2014) Turner syndrome revisited: review of new data supports the hypothesis that all viable 45,X cases are cryptic mosaics with a rescue cell line, implying an origin by mitotic loss. Hum Genet 133: 417-424.

8. Benn PA, Ying J. (2004) Preliminary estimate for the second-trimester maternal serum screening detection rate of the 45,X karyotype using alpha-fetoprotein, unconjugated estriol and human chorionic gonadotropin. J Matern Fetal Neonatal Med 15: 160-166.

9. Wald NJ, Rodeck C, Hackshaw AK, Walters J, Chitty L, Mackinson AM. (2003) First and second trimester antenatal screening for Down's syndrome: the results of the Serum, Urine and Ultrasound Screening Study (SURUSS). J Med Screen 10: 56-104.

10. Wald NJ, Rodeck C, Hackshaw AK, Walters J, Chitty L, Mackinson AM. (2006) First and second trimester antenatal screening for Down's syndrome: the results of the Serum, Urine and Ultrasound Screening Study (SURUSS). Erratum. J Med Screen 13: 51-52.

11. Wald N, Rodeck C, Rudnicka A, Hackshaw A. (2004) Nuchal translucency and gestational age. Prenat Diagn 24: 150-151.

12. Egan JF, Smith K, Timms D, Bolnick JM, Campbell WA, Benn PA. (2011) Demographic differences in Down syndrome livebirths in the US from 1989 to 2006. Prenat Diagn 31: 389-394.

13. Benn P, Wright D, Cuckle H. (2005) Practical strategies in contingent sequential screening for Down syndrome. Prenat Diagn 25: 645-652.

14. Tul N, Spencer K, Noble P, Chan C, Nicolaides K. (1999) Screening for trisomy 18 by fetal nuchal translucency and maternal serum free beta-hCG and PAPP-A at 10-14 weeks of gestation. Prenat Diagn 19: 1035-1042.

15. Palomaki GE, Neveux LM, Knight GJ, Haddow JE. (2003) Maternal serum-integrated screening for trisomy 18 using both first- and second-trimester markers. Prenat Diagn 23: 243-247.

16. Palomaki GE, Haddow JE, Knight GJ, Wald NJ, Kennard A, Canick JA, et al. (1995) Risk-based prenatal screening for trisomy 18 using alpha-fetoprotein, unconjugated oestriol and human chorionic gonadotropin. Prenat Diagn 15: 713-723.

17. Spencer K, Nicolaides KH. (2002) A first trimester trisomy 13/trisomy 18 risk algorithm combining fetal nuchal translucency thickness, maternal serum free beta-hCG and PAPP-A. Prenat Diagn 22: 877-879.

18. Spencer K, Tul N, Nicolaides KH. (2000) Maternal serum free beta-hCG and PAPP-A in fetal sex chromosome defects in the first trimester. Prenat Diagn 20: 390-394.

19. Beamon CJ, Hardisty EE, Harris SC, Vora NL. (2014) A single center's experience with noninvasive prenatal testing. Genet Med 27: 681–687.

20. Lau TK, Cheung SW, Lo PS, Pursley AN, Chan MK, Jiang F, et al. (2014) Non-invasive prenatal testing for fetal chromosomal abnormalities by low-coverage whole-genome sequencing of maternal plasma DNA: review of 1982 consecutive cases in a single center. Ultrasound Obstet Gynecol 43: 254-264.

21. Fairbrother G, Johnson S, Musci TJ, Song K. (2013) Clinical experience of noninvasive prenatal testing with cell-free DNA for fetal trisomies 21, 18, and 13, in a general screening population. Prenat Diagn 33: 580-583.

22. Gil MM, Quezada MS, Bregant B, Ferraro M, Nicolaides KH. (2013) Implementation of maternal blood cell-free DNA testing in early screening for aneuploidies. Ultrasound Obstet Gynecol 42: 34-40.

23. Comas C, Echevarria M, Rodriguez MA, Prats P, Rodriguez I, Serra B. (2014) Initial experience with non-invasive prenatal testing of cell-free DNA for major chromosomal anomalies in a clinical setting. J Matern Fetal Neonatal Med 12: 1-6.

24. Dan S, Wang W, Ren J, Li Y, Hu H, Xu Z, et al. (2012) Clinical application of massively parallel sequencing-based prenatal noninvasive fetal trisomy test for trisomies 21 and 18 in 11,105 pregnancies with mixed risk factors. Prenat Diagn 32: 1225-1232.

25. Futch T, Spinosa J, Bhatt S, de Feo E, Rava RP, Sehnert AJ. (2013) Initial clinical laboratory experience in noninvasive prenatal testing for fetal aneuploidy from maternal plasma DNA samples. Prenat Diagn 33: 569-574.

26. Dar P, Curnow KJ, Gross SJ, Hall MP, Stosic M, Demko Z, et al. (2014) Clinical experience and follow-up with large scale single-nucleotide polymorphism-based non-invasive prenatal aneuploidy testing. Am J Obstet Gynecol 211: 527.e1-17.

27. Sparks AB, Struble CA, Wang ET, Song K, Oliphant A. (2012) Noninvasive prenatal detection and selective analysis of cell-free DNA obtained from maternal blood: evaluation for trisomy 21 and trisomy 18. Am J Obstet Gynecol 206: 319.e1-9.

28. Hall MP, Hill M, Zimmermann B, Sigurjonsson S, Westemeyer M, Saucier J, et al. (2014) Non-invasive prenatal detection of trisomy 13 using a single nucleotide polymorphism- and informatics-based approach. PLoS One 9: e96677.

29. Samango-Sprouse C, Banjevic M, Ryan A, Sigurjonsson S, Zimmermann B, Hill M, et al. (2013) SNP-based non-invasive prenatal testing detects sex chromosome aneuploidies with high accuracy. Prenat Diagn 33: 643-649.

30. American Congress of Obstetricians and Gynecologists Committee on Genetics (2012) Committee Opinion No. 545: Noninvasive prenatal testing for fetal aneuploidy. Obstet Gynecol 120: 1532-1534.

31. Shaffer BL, Caughey AB, Norton ME. (2006) Variation in the decision to terminate pregnancy in the setting of fetal aneuploidy. Prenat Diagn 26: 667-671.

32. Palomaki GE, Knight GJ, Ashwood ER, Best RG, Haddow JE. (2013) Screening for down syndrome in the United States: results of surveys in 2011 and 2012. Arch Pathol Lab Med 137: 921-926.

33. Shah FT, French KS, Osann KE, Bocian M, Jones MC, Korty L. (2014) Impact of cell-free fetal DNA screening on patients’ choice of invasive procedures after a positive California Prenatal Screen result. J Clin Med 3: 849-864.

34. Blumenfeld YJ, Taylor J, Lee HC, Hudgins L, Sung JF, El-Sayed YY. (2012) Utilization of available prenatal screening and diagnosis: effects of the California screen program. J Perinatol 32: 907-912.

35. Tabor A, Alfirevic Z. (2010) Update on procedure-related risks for prenatal diagnosis techniques. Fetal Diagn Ther 27: 1-7.

36. Song K, Musci TJ, Caughey AB. (2013) Clinical utility and cost of non-invasive prenatal testing with cfDNA analysis in high-risk women based on a US population. J Matern Fetal Neonatal Med 26: 1180-1185.

37. Shamshirsaz AA, Ravangard SF, Turner G, Borgida A, Janicki MB, Campbell WA, et al. (2013) Efficacy of the genetic sonogram in a stepwise sequential protocol for down syndrome screening. J Ultrasound Med 32: 1607-1613.

38. Fang YM, Benn P, Campbell W, Bolnick J, Prabulos AM, Egan JF. (2009) Down syndrome screening in the United States in 2001 and 2007: a survey of maternal-fetal medicine specialists. Am J Obstet Gynecol 201: 97.e1-5.

39. Gregg AR, Gross SJ, Best RG, Monaghan KG, Bajaj K, Skotko BG, et al. (2013) ACMG statement on noninvasive prenatal screening for fetal aneuploidy. Genet Med 15: 395-398.

40. Waitzman NJ, Romano PS, Scheffler RM. (1994) Estimates of the economic costs of birth defects. Inquiry 31: 188-205.

41. Agency for Healthcare Research and Quality (2006 and 2009) Healthcare Cost and Utilization Project (HCUP) Kids’ Inpatient Database (KID). Available: <http://www.hcup-us.ahrq.gov/overview.jsp> Accessed 23 December 2014.

42. Saenger P, Wikland KA, Conway GS, Davenport M, Gravholt CH, Hintz R, et al. (2001) Recommendations for the diagnosis and management of Turner syndrome. J Clin Endocrinol Metab 86: 3061-3069.
