# Supplementary material for: An Economic Analysis of Cell-Free DNA Non-Invasive Prenatal Testing in the US General Pregnancy Population
Source: PLoS One. 2015 Jul 9;10(7):e0132313. doi: 10.1371/journal.pone.0132313 (PMC4497716; doi:10.1371/journal.pone.0132313)
Supplement: S1 Table — (DOCX) [file pone.0132313.s002.docx]

**Supporting Table 1. Baseline cost variables**

| **Model Component** | **Cost (2014 USD)** | **Reference** |
| --- | --- | --- |
| *Clinical Test/Clinical Care* |  |  |
| Amniocentesis | $835 | [43,44] |
| Chorionic villus sampling (CVS) | $892 | [43,44] |
| Second trimester screening | $136 | [43,44] |
| Sequential screening | $136 | [43,44] |
| Genetic Counseling | $56 | [43] |
| Prenatal care | $6,552 | [43,44] |
| Ultrasound | $168 | [43,44] |
| Office Visit | $96 | [43] |
| NT Charge | $147 | [43,44] |
| Papp A | $25 | [44] |
| HCG | $25 | [44] |
| Genetic sonogram | $223 | [43,44] |
| HCG | $25 | [44] |
| AFP | $27 | [44] |
| Unconjugated estriol (uE3) | $40 | [44] |
| Inhibin A | $26 | [44] |
| *Cost of elective termination* |  |  |
| Early, following CVS | $562 | [45-49] |
| Late, following amniocentesis | $2,997 | [45,46,48,49] |
|  | | |

**References**

43. Centers for Medicare and Medicaid Services. Physician Fee Schedule. 2014.

44. Centers for Medicare and Medicaid Services. Clinical Diagnostic Laboratory Fee Schedule. 2014.

45. Biggio JR, Jr., Morris TC, Owen J, Stringer JS. (2004) An outcomes analysis of five prenatal screening strategies for trisomy 21 in women younger than 35 years. Am J Obstet Gynecol 190: 721-729.

46. US Bureau of Labor Statistics. Consumer price indices. 2014. Washington, DC.

47. Ohno M, Caughey A. (2013) The role of noninvasive prenatal testing as a diagnostic versus a screening tool--a cost-effectiveness analysis. Prenat Diagn 33: 630-635.

48. Odibo AO, Stamilio DM, Nelson DB, Sehdev HM, Macones GA. (2005) A cost-effectiveness analysis of prenatal screening strategies for Down syndrome. Obstet Gynecol 106: 562-568.

49. Caughey AB, Kaimal AJ, Odibo AO. (2010) Cost-effectiveness of Down syndrome screening paradigms. Clin Lab Med 30: 629-642.
