# Supplementary material for: An Economic Analysis of Cell-Free DNA Non-Invasive Prenatal Testing in the US General Pregnancy Population
Source: PLoS One. 2015 Jul 9;10(7):e0132313. doi: 10.1371/journal.pone.0132313 (PMC4497716; doi:10.1371/journal.pone.0132313)
Supplement: S2 Table — (DOCX) [file pone.0132313.s003.docx]

**Supporting Table 2. Assay performance with cfDNA NIPT**

| **Reference** | **Method** | **Trisomy 21** | | | | **Trisomy 18** | | | | **Trisomy 13** | | | | **Monosomy X** | | | |
| --- | --- | --- | --- | --- | --- | --- | --- | --- | --- | --- | --- | --- | --- | --- | --- | --- | --- |
|  |  | **TP** | **FN** | **FP** | **TN** | **TP** | **FN** | **FP** | **TN** | **TP** | **FN** | **FP** | **TN** | **TP** | **FN** | **FP** | **TN** |
| Chiu *et al.* 2011 [50] | s-MPS | 86 | 0 | 3 | 143 | - | - | - | - | - | - | - | - | - | - | - | - |
| Ehrich *et al.* 2011 [51] | s-MPS | 39 | 0 | 1 | 409 | - | - | - | - | - | - | - | - | - | - | - | - |
| Palomaki *et al.* 2011 [52] | s-MPS | 209 | 3 | 3 | 1,468 | - | - | - | - | - | - | - | - | - | - | - | - |
| Palomaki et al. 2012 [53] | s-MPS | - | - | - | - | 59 | 0 | 5 | 1,683 | 11 | 1 | 16 | 1,672 | - | - | - | - |
| Porreco *et al.* 2014 [54] | s-MPS | 137 | 0 | 3 | 3,182 | 36 | 3 | 0 | 3,283 | 14 | 2 | 0 | 3,306 | 9 | 0 | 11 | 3,258 |
| Mazloom *et al.* 2013 [55] | s-MPS | - | - | - | - | - | - | - | - | - | - | - | - | 17 | 1 | 1 | 392 |
| Bianchi *et al.* 2012 [56] | s-MPS | 89 | 1 | 0 | 410 | 35 | 3 | 0 | 463 | 11 | 5 | 0 | 485 | 15 | 5 | 1 | 461 |
| Bianchi *et al.* 2014 [57] | s-MPS | 5 | 0 | 6 | 1,898 | 2 | 0 | 3 | 1,900 | 1 | 0 | 3 | 1,910 | - | - | - | - |
| Liang *et al.* 2013 [58] | s-MPS | 40 | 0 | 0 | 372 | 14 | 0 | 0 | 398 | 4 | 0 | 1 | 407 | 5 | 0 | 1 | 406 |
| Song *et al.* 2013 [59] | s-MPS | 8 | 0 | 0 | 1,733 | 2 | 0 | 1 | 1,738 | 1 | 0 | 0 | 1,740 | 2 | 1 | 0 | 1,737 |
| Stumm *et al.* 2014 [60] | s-MPS | 40 | 1 | 0 | 430 | 8 | 0 | 1 | 462 | 5 | 0 | 0 | 466 | - | - | - | - |
| Ashoor *et al.* 2012 [61] | t-MPS | 50 | 0 | 0 | 297 | 49 | 1 | 0 | 297 | - | - | - | - | - | - | - | - |
| Norton *et al.* 2012 [62] | t-MPS | 81 | 0 | 1 | 2,887 | 37 | 1 | 2 | 2,886 | - | - | - | - | - | - | - | - |
| Ashoor *et al.* 2013 [63] | t-MPS | - | - | - | - | - | - | - | - | 8 | 2 | 1 | 1,938 | - | - | - | - |
| Verweij *et al.* 2013 [64] | t-MPS | 17 | 1 | 0 | 486 | - | - | - | - | - | - | - | - | - | - | - | - |
| Nicolaides *et al.* 2012 [65] | t-MPS | 8 | 0 | 0 | 1,941 | 2 | 0 | 2 | 1,945 | - | - | - | - | - | - | - | - |
| Nicolaides *et al.* 2014 [66] | t-MPS | - | - | - | - | - | - | - | - | - | - | - | - | 43 | 4 | 0 | 125 |
| Nicolaides *et al.* 2013 [67] | SNP | 25 | 0 | 0 | 204 | 3 | 0 | 0 | 226 | 1 | 0 | 0 | 228 | 2 | 0 | 0 | 227 |
| Pergament *et al.* 2014 [68] | SNP | 58 | 0 | 0 | 905 | 24 | 1 | 1 | 938 | 12 | 0 | 0 | 953 | 9 | 1 | 1 | 954 |
| TOTAL | | 892 | 6 | 17 | 16,765 | 271 | 9 | 15 | 16,219 | 68 | 10 | 21 | 13,105 | 102 | 12 | 15 | 7,560 |
| **Sensitivity** | | **99.33%** (892/898) | | | | **96.79%** (271/280) | | | | **87.18%** (68/78) | | | | **89.47%** (102/114) | | | |
| **Specificity** | | **99.90%** (16,765/16,782) | | | | **99.91%** (16,219/16,234) | | | | **99.84%** (13,105/13,126) | | | | **99.80%** (7,560/7,575) | | | |

True Positive (TP); False Negative (FN); False Positive (FP); True Negative (TN); Shotgun massively parallel sequencing (s-MPS); targeted massively parallel sequencing (t-MPS); Single-nucleotide polymorphism (SNP)-based sequencing.

**References**

50. Chiu RW, Akolekar R, Zheng YW, Leung TY, Sun H, Chan KC, et al. (2011) Non-invasive prenatal assessment of trisomy 21 by multiplexed maternal plasma DNA sequencing: large scale validity study. BMJ 342: c7401.

51. Ehrich M, Deciu C, Zwiefelhofer T, Tynan JA, Cagasan L, Tim R, et al. (2011) Noninvasive detection of fetal trisomy 21 by sequencing of DNA in maternal blood: a study in a clinical setting. Am J Obstet Gynecol 204: 205.e1-11.

52. Palomaki GE, Kloza EM, Lambert-Messerlian GM, Haddow JE, Neveux LM, Ehrich M, et al. (2011) DNA sequencing of maternal plasma to detect Down syndrome: an international clinical validation study. Genet Med 13: 913-920.

53. Palomaki GE, Deciu C, Kloza EM, Lambert-Messerlian GM, Haddow JE, Neveux LM, et al. (2012) DNA sequencing of maternal plasma reliably identifies trisomy 18 and trisomy 13 as well as Down syndrome: an international collaborative study. Genet Med 14: 296-305.

54. Porreco RP, Garite TJ, Maurel K, Marusiak B, Ehrich M, van den Boom D, et al. (2014) Noninvasive prenatal screening for fetal trisomies 21, 18, 13 and the common sex chromosome aneuploidies from maternal blood using massively parallel genomic sequencing of DNA. Am J Obstet Gynecol 211: 365.e1-12.

55. Mazloom AR, Dzakula Z, Oeth P, Wang H, Jensen T, Tynan J, et al. (2013) Noninvasive prenatal detection of sex chromosomal aneuploidies by sequencing circulating cell-free DNA from maternal plasma. Prenat Diagn 33: 591-597.

56. Bianchi DW, Platt LD, Goldberg JD, Abuhamad AZ, Sehnert AJ, Rava RP. (2012) Genome-wide fetal aneuploidy detection by maternal plasma DNA sequencing. Obstet Gynecol 119: 890-901.

57. Bianchi DW, Parker RL, Wentworth J, Madankumar R, Saffer C, Das AF, et al. (2014) DNA sequencing versus standard prenatal aneuploidy screening. N Engl J Med 370: 799-808.

58. Liang D, Lv W, Wang H, Xu L, Liu J, Li H, et al. (2013) Non-invasive prenatal testing of fetal whole chromosome aneuploidy by massively parallel sequencing. Prenat Diagn 33: 409-15.

59. Song Y, Liu C, Qi H, Zhang Y, Bian X, Liu J. (2013) Noninvasive prenatal testing of fetal aneuploidies by massively parallel sequencing in a prospective Chinese population. Prenat Diagn 33: 700-706.

60. Stumm M, Entezami M, Haug K, Blank C, Wustemann M, Schulze B, et al. (2014) Diagnostic accuracy of random massively parallel sequencing for non-invasive prenatal detection of common autosomal aneuploidies: a collaborative study in Europe. Prenat Diagn 34: 185-191.

61. Ashoor G, Syngelaki A, Wagner M, Birdir C, Nicolaides KH. (2012) Chromosome-selective sequencing of maternal plasma cell-free DNA for first-trimester detection of trisomy 21 and trisomy 18. Am J Obstet Gynecol 206: 322.e1-5.

62. Norton ME, Brar H, Weiss J, Karimi A, Laurent LC, Caughey AB, et al. (2012) Non-Invasive Chromosomal Evaluation (NICE) Study: results of a multicenter prospective cohort study for detection of fetal trisomy 21 and trisomy 18. Am J Obstet Gynecol 207: 137.e1-8.

63. Ashoor G, Syngelaki A, Wang E, Struble C, Oliphant A, Song K, et al. (2013) Trisomy 13 detection in the first trimester of pregnancy using a chromosome-selective cell-free DNA analysis method. Ultrasound Obstet Gynecol 41: 21-25.

64. Verweij EJ, Jacobsson B, van Scheltema PA, de Boer MA, Hoffer MJ, Hollemon D, et al. (2013) European Non-Invasive Trisomy Evaluation (EU-NITE) study: a multicenter prospective cohort study for non-invasive fetal trisomy 21 testing. Prenat Diagn 33: 996-1001.

65. Nicolaides KH, Syngelaki A, Ashoor G, Birdir C, Touzet G. (2012) Noninvasive prenatal testing for fetal trisomies in a routinely screened first-trimester population. Am J Obstet Gynecol 207: 374.e1-6.

66. Nicolaides KH, Musci TJ, Struble CA, Syngelaki A, Gil MM. (2014) Assessment of fetal sex chromosome aneuploidy using directed cell-free DNA analysis. Fetal Diagn Ther 35: 1-6.

67. Nicolaides KH, Syngelaki A, Gil M, Atanasova V, Markova D. (2013) Validation of targeted sequencing of single-nucleotide polymorphisms for non-invasive prenatal detection of aneuploidy of chromosomes 13, 18, 21, X, and Y. Prenat Diagn 33: 575-579.

68. Pergament E, Cuckle H, Zimmermann B, Banjevic M, Sigurjonsson S, Ryan A, et al. (2014) Single-nucleotide polymorphism-based noninvasive prenatal screening in a high-risk and low-risk cohort. Obstet Gynecol 124: 210-218.
